# The Week in the Courts

**Published:** 1910-12-24

**Authors:** 


### The Week in the Courts.

### TREATMENT OF INEBRIATES IN SCOTLAND.

According to the report of the Inspector for Scotland under the Inebriates Acts for the year 1909, the total number of persons dealt with during that period was 239, of whom 121 were retreat patients and 118 reformatory inmates. The "recovery rate" in well-conducted retreats is found to approach 50 per cent., and in reformatories about 7 per cent. The total accommodation in licensed retreats and in inebriate reformatories in Scotland amounts to 229 beds: 74 in retreats, 116 in certified reformatories, and 39 in the State Reformatory.—" Report of Inspector under the Inebriates Act (Scotland)," Cd. 5364, price 3d.

### THE SALE OF POISONS.

Mr. Justice Phillimore and Mr. Justice Horridge, sitting as a Divisional Court, have decided an important question arising out of the interpretation of the Poisons and Pharmacy Act, 1908. Under section 2 of that Act licences have been granted to ironmongers, seedsmen, etc., to sell preparations containing arsenic and nicotine for use in agriculture and horticulture. In the view of the Pharmaceutical Society only the licensee himself is entitled to sell poisons, and the Society instituted proceedings against the assistant to a seedsman who sold poisons, claiming that he was covered by his master's licence. The Kingston-on-Thames County Court Judge upheld the Society's view, and ordered the assistant to pay the penalty. On appeal to a Divisional Court this decision was upheld, and the appeal dismissed. The unlicensed assistant to a licensed poison-seller is thus in the same position as the unqualified chemist's assistant, who may not sell poisons.

